# A New Test Statistic Based on Shrunken Sample Variance for Identifying Differentially Expressed Genes in Small Microarray Experiments

**DOI:** 10.4137/bbi.s473

**Published:** 2008-02-29

**Authors:** Akihiro Hirakawa, Yasunori Sato, Chikuma Hamada, Isao Yoshimura

**Affiliations:** 1 Genetics Division, National Cancer Center Research Institute, Chuo-ku, Tokyo, Japan; 2 Department of Biostatistics, Harvard School of Public Health, Boston, U.S.A; 3 Faculty of Engineering, Tokyo University of Science, Shinjuku-ku, Tokyo, Japan

**Keywords:** differentially expressed genes, false discovery rate, microarray, shrunken sample variance, significance analysis of microarray, t-type score

## Abstract

Choosing an appropriate statistic and precisely evaluating the false discovery rate (FDR) are both essential for devising an effective method for identifying differentially expressed genes in microarray data. The *t*-type score proposed by Pan et al. (2003) succeeded in suppressing false positives by controlling the underestimation of variance but left the overestimation uncontrolled. For controlling the overestimation, we devised a new test statistic (variance stabilized *t*-type score) by placing shrunken sample variances of the James-Stein type in the denominator of the *t*-type score. Since the relative superiority of the *mean* and *median FDR*s was unclear in the widely adopted Significance Analysis of Microarrays (SAM), we conducted simulation studies to examine the performance of the variance stabilized *t*-type score and the characteristics of the two FDRs. The variance stabilized *t*-type score was generally better than or at least as good as the *t*-type score, irrespective of the sample size and proportion of differentially expressed genes. In terms of accuracy, the *median FDR* was superior to the *mean FDR* when the proportion of differentially expressed genes was large. The variance stabilized *t*-type score with the *median FDR* was applied to actual colorectal cancer data and yielded a reasonable result.

## Introduction

In recent biological studies, the development of DNA microarray technology allows simultaneous measurement of the expression levels of thousands of genes and identification of genes that are differentially expressed across cells or tissues under different conditions such as normal or disease conditions. Identifying differentially expressed genes poses complex multiple testing problems and reduces the power for statistical tests because thousands of genes are evaluated simultaneously in typical microarray experiments. In such cases, it is difficult to precisely identify differentially expressed genes using traditional statistical methods, and this difficulty is exacerbated when the sample size is small.

The traditional test statistics for comparing the gene expression levels under different conditions are the Student’s *t*-statistic, Welch *t*-statistic, and Mann-Whitney statistic. These test statistics have been used since the beginning of microarray experiments, although their performances have not been sufficiently evaluated. After that, many test statistics suitable for microarray data have been proposed, i.e. Golub’s discrimination score (Golub et al. 1999), the SAM-statistic (Tusher et al. 2001), *t*-type score, and samroc statistic (Broberg, 2003). Golub’s discrimination score is a statistic wherein the mean difference between two groups is divided by the sum of the standard deviations of both groups. The SAM statistic is a modified Student’s *t*-statistic with a correction term added to its denominator. The samroc statistic is a ROC-based SAM statistic whose performance is almost the same as that of the SAM-statistic. Recently, some new test statistics have been proposed (Chen et al. 2007; Opgen-Rhein and Strimmer, 2007; Sartor et al. 2006).

Choosing an appropriate test statistic is essential for devising an effective method for identifying differentially expressed genes. We need to suppress both false positives, which are genes that are incorrectly identified as differentially expressed genes, and false negatives, which are genes that are incorrectly identified as nondifferentially expressed genes. Both false positives and false negatives mainly arise due to the underestimation and overestimation of the variance of gene expression levels, respectively. The Student’s *t*-statistic, Welch *t*-statistic, and Golub’s discrimination score leave both the underestimation and overestimation of variance uncontrolled, resulting in an increased risk of both false positives and false negatives (Broberg, 2003; Pan, 2002). The SAM-statistic and *t-*type score (Pan et al. 2003) with a correction term added to the denominator of the Welch *t*-statistic can suppress false positives by controlling the underestimation of variance, but they leave the overestimation uncontrolled. To control the overestimation, we devised a new test statistic (variance stabilized *t*-type score) by placing shrunken sample variances of the James-Stein type (Cui et al. 2005; Stein, 1956; Stein, 1964) in the denominator of the *t*-type score. The shrunken sample variances, which can borrow information across genes using the James-Stein shrinkage concept, can control the overestimation of variance. The variance stabilized *t*-type score can suppress both false positives and false negatives by adding the correction term and placing the shrunken sample variances, respectively.

According to the article published by Dupuy and Simon (2007), from among the many test statistics, the SAM statistic, Golub’s discrimination score, the *t*-statistic, and the Mann-Whitney statistic have been frequently used in actual microarray data analysis. We, therefore, conducted a simulation study to compare the performances of the Mann-Whitney statistic, Golub’s discrimination score, the Welch *t*-statistic, the *t*-type score, and the variance stabilized *t*-type score in small microarray experiments (Simulation study 1).

The false discovery rate (FDR) introduced by Benjamini and Hochberg (1995) as a criterion for optimizing the identifiability of differentially expressed genes is also essential for devising an effective method. The FDR is defined as the expected proportion of false positives among total positives, which represent all identified genes. Using the FDR, we can identify differentially expressed genes using a cut-off value corresponding to the target FDR, e.g. FDR <0.05. Recently, many FDR estimation methods have been proposed, such as Significance Analysis of Microarrays (SAM) (Tusher et al. 2001), the empirical Bayes (EB) method (Efron, 2001), and the mixture model method (MMM) (Pan et al. 2003). Among these, SAM is widely used (Dupuy and Simon, 2007) and estimates the number of false positives based on a permutation procedure. Note that SAM provides two FDRs, i.e. the *mean FDR* and *median FDR*. In the original description of SAM, the *mean FDR* is obtained by using the mean of the estimated number of false positives in each permutation, while the SAM software in the R package (Chu et al. 2005) provides the *median FDR* using the median of the estimated number of false positives in each permutation. Although the values of both FDRs are quite different in the actual microarray data analysis, the relative superiority of the two FDRs is unclear. Therefore, we examined the accuracy of both the *mean* and the *median FDR*s by using SAM in small microarray experiments (Simulation study 2).

Additionally, SAM using the variance stabilized *t*-type score was applied to colorectal cancer data comprising approximately 22,000 probesets of 3 cells from the primary tumor and 3 cells from a lymph node metastasis (http://www.ncbi.nlm.nih.gov/projects/geo/gds/; Provenzani et al. 2006) in order to demonstrate the practical application of both FDRs using the variance stabilized *t*-type score.

## Materials and Methods

### Test statistics

For each gene *i*, *i* = 1, 2,…, *g*, the expression level is *X_i_*_1_, …, *X_im_* from *m* samples collected from cells or tissues under Condition 1, and it is *Y_i_*_1_, …, *Y_in_* from *n* samples collected from cells or tissues under Condition 2. *X_i_*_1_, …, *X_im_* are normal random variables with true mean *μ_Xi_* and true variance σ*_Xi_*^2^, and *Y_i_*_1_, …, *Y_in_* are normal random variables with true mean *μ_Yi_* and true variance σ*_Yi_*^2^. If the true means of the two conditions are different, the gene is defined as being differentially expressed, and if the true means of the two conditions are the same, the gene is defined as being nondifferentially expressed.

The Mann-Whitney statistic is a nonparametric test statistic that does not assume specific distributions. For gene *i*, all the expression levels are ranked without regard to which sample they are in, being arranged into a single ranked series. Let *u_i_* denote the Mann-Whitney statistic for gene *i; u_i_* can be written as

(1) $$u_{i}=\frac{12mn{\left({\bar{R}}_{Xi}-{\bar{R}}_{Yi}\right)}^{2}}{{\left(m+n\right)}^{2}(m+n+1)f_{i}},$$

where *R̄_Xi_* is the mean rank of *m* samples in Condition 1, and *R̄_Yi_* is the mean rank of *n* samples in Condition 2. Also, let *T* and *t_k_* be the size of tie expression levels in both conditions and the number of *k*th tie expression levels, respectively; *f_i_* can be written as *f_i_* = 1 − ∑*_k_*_=1_*^T^* *t_k_* (*t_k_* − 1)(*t_k_* + 1)/(*m* + *n*)(*m* + *n* − 1) (*m* + *n* + 1).

Golub’s discrimination score is a test statistic that is similar to the Welch *t*-statistic. Let *d_i_* denote Golub’s discrimination score for gene *i; d_i_* can be written as

(2) $$d_{i}=\frac{{\bar{X}}_{i}-{\bar{Y}}_{i}}{\sqrt{s_{Xi}^{2}}+\sqrt{s_{Yi}^{2}}},$$

where *X̄_i_* = ∑*_j=_*_1_*^m^* *X_ij_*/*m* and *X̄_i_* = ∑*_j=_*_1_*^n^* *Y_ij_*/*n* are the sample means for gene *i* under Conditions 1 and 2, respectively, and *s_Xi_*^2^ = ∑*_j=_*_1_*^m^* (*X_ij_* − *X̄_i_*)^2^/(*m* − 1) and *s_Yi_*^2^ = ∑*_j=_*_1_*^n^* (*Y_ij_* − *X̄_i_*)^2^/(*n* − 1) are the sample variances for gene *i* under Conditions 1 and 2, respectively.

The Welch *t-*statistic is a typical test statistic for comparing gene expression levels under two conditions. Let *w_i_* denotes the Welch *t-*statistic for gene *i; w_i_* can be written as

(3) $$w_{i}=\frac{{\bar{X}}_{i}-{\bar{Y}}_{i}}{\sqrt{s_{Xi}^{2}/m+s_{Yi}^{2}/n}}.$$

In the case where the Welch *t*-statistic is used, since thousands of genes are evaluated simultaneously, when some of them have underestimated sample variance under two conditions by chance, their absolute test statistic becomes large even though their mean difference is not meaningfully large. In such cases, the total positives contain many false positives. For suppressing false positives, the *t*-type score with a correction term added to the denominator of the Welch *t*-statistic has been proposed based on the basic idea of the SAM-statistic. Let *z_i_* denote the *t*-type score for gene *i; z_i_* can be written as

(4) $$z_{i}=\frac{{\bar{X}}_{i}-{\bar{Y}}_{i}}{\sqrt{s_{Xi}^{2}/m+s_{Yi}^{2}/n}+a_{0}},$$

where *a*_0_ is a correction term and is the 90th percentile of $\left\{\sqrt{s_{Xi}^{2}/m+s_{Yi}^{2}/n}:i=1,\ldots ,g\right\}$. A correction term serves as a control for the underestimation of variance, and the *t*-type score can suppress false positives when a correction term is used. However, the *t*-type score leaves the overestimation of variance uncontrolled, resulting in an increased risk of false negatives.

We devised the variance stabilized *t*-type score by placing shrunken sample variances of the James-Stein type in the denominator of the *t*-type score to control the overestimation of variance. Let *v_i_* denotes the variance stabilized *t*-type score for gene *i; v_i_* can be written as

(5) $$v_{i}=\frac{{\bar{X}}_{i}-{\bar{Y}}_{i}}{\sqrt{{\tilde{s}}_{Xi}^{2}/m+{\tilde{s}}_{Yi}^{2}/n}+{\tilde{a}}_{0}},$$

Where ${\tilde{s}}_{Xi}^{2}=s_{Xi}^{2}-\frac{s_{Xi}^{2}}{s_{Xi}^{2}+{\bar{s}}_{X}^{2}}(s_{Xi}^{2}-{\bar{s}}_{X}^{2}),{\bar{s}}_{X}^{2}=\Sigma_{i=1}^{g}s_{Xi}^{2}/g$ and ${\tilde{s}}_{Yi}^{2}=s_{Yi}^{2}-\frac{s_{Yi}^{2}}{s_{Yi}^{2}+{\bar{s}}_{Y}^{2}}(s_{Yi}^{2}-{\bar{s}}_{Y}^{2}),{\bar{s}}_{Y}^{2}=\Sigma_{i=1}^{g}s_{Yi}^{2}/g$ are the shrunken sample variances for gene *i* under two conditions, respectively, and *ã*_0_ is the 90th percentile of $\left\{\sqrt{{\tilde{s}}_{Xi}^{2}/m+{\tilde{s}}_{Yi}^{2}/n}:i=1,\ldots ,g\right\}$. Based on Equation (5), when *s_Xi_*^2^ (*s_Yi_*^2^) is larger than *s̄_X_*^2^ (*s̄_Y_*^2^), *s_Xi_*^2^ (*s_Yi_*^2^) is shrunken toward *s̄_X_*^2^ (*s̄_Y_*^2^). On the other hand, when *s_Xi_*^2^ (*s_Yi_*^2^) is smaller than *s̄_X_*^2^ (*s̄_Y_*^2^), *s_Xi_*^2^ (*s_Y_*^2^) is not shrunken toward *s̄_X_*^2^ (*s̄_Y_*^2^) and remains as it is. These behaviors of shrunken sample variances control the overestimation of variance. After controlling the overestimation, a correction term controls the underestimation of variance as well as the *t*-type score. Thus, the variance stabilized *t*-type score can suppress false positives and false negatives, simultaneously.

### False discovery rate

The FDR is a popular criterion for identifying differentially expressed genes in microarray data analysis. In this paper, we use the FDR definition given by Storey and Tibshirani (2003). For a fixed cut-off value, *c*, for a test statistic, we can obtain the true FDR and its estimator as

(6) $$FDR(c)=\frac{\pi_{0}FP(c)}{TP(c)},\;\;\;F\hat{D}R(c)=\frac{{\hat{\pi }}_{0}\hat{F}P(c)}{\hat{T}P(c)},$$

where π_0_ is the proportion of true nondifferentially expressed genes among the total candidate genes, and π̂_0_ is its estimator. For a fixed cut-off value, *c*, *FP*(*c*) is defined as the number of true false positives, and *F̂P*(*c*) is defined as the estimated number of false positives. Similarly, we define *T̂P*(*c*) as the estimated number of total positives. This definition of the FDR has been widely used in recent microarray data analysis, although the FDR is defined as *F̂P*(*c*)/*T̂P*(*c*) in the original SAM description. Based on Equation (6), it is necessary to estimate the proportion of true nondifferentially expressed genes, π_0_, in order to calculate the FDR. Although many statistical methods have been proposed in recent studies to estimate π_0_ (Chu et al. 2005; Efron et al. 2001; Lee et al. 2000; Storey, 2002), the precise estimation is very difficult (Xie et al. 2005) and is itself an unresolved research problem. Since our study does not focus on the estimation of π_0_, we use true π_0_ in the simulation study. However, in the actual data analysis, we estimate π_0_ using the method of Chu et al. (2005).

### *Mean* and *median FDR*s

For calculating the estimated number of total positives, we calculate any test statistic *t_i_* for gene *i*, *i* = 1, …, *g*, from raw data, corresponding to the ordered statistics *t*_(1)_ ≤ *t*_(2)_ … ≤ *t*_(_*_g_*_)_. For the fixed cut-off value, *c*, we identify gene *i* that satisfies | *t*_(_*_i_*_)_| > *c* as a differentially expressed gene. The estimated number of total positives is defined as *T̂P*(*c*) = #{*i*: | *t*_(_*_i_*_)_| > *c*}.

We also need to estimate the number of false positives (*FP*). The SAM estimates the number of false positives based on the permutation from all samples in a total of *B* times. For the *b*th permutated data, we calculate the test statistics *t_i_^b^*, *b* = 1, …, *B* and *i* = 1, …, *g*, and corresponding ordered statistics *t*_(1)_*^b^* ≤ *t*_(2)_*^b^* ≤ *t*_(_*_g_*_)_*^b^*. After the permutation, we obtain the number of genes that satisfy | *t*_(_*_i_*_)_*^b^* | > *c*, #{*i*: | *t̄*_(_*_i_*_)_*^b^* | > *c*}, *i* = 1, …, *g*, in each permutation. The mean of these numbers of genes is defined as the *mean F̂P*(*c*), and the median of these numbers of genes is defined as the *median F̂P*(*c*). The *mean F̂P*(*c*) and *median F̂P*(*c*) can be represented as follows:

(7) $$mean\;\hat{F}P(c)=1/B\sum_{b=1}^{B}\#\{i:|t_{\left(i\right)}^{b}|\;>c\}$$

and

(8) $$\begin{array}{l} median\;\hat{F}P(c)=median(\#\{i:|t_{\left(i\right)}^{1}|\;>c\},\\ \#\{i:|t_{\left(i\right)}^{2}|\;>c\},\ldots ,\#\{i:|t_{\left(i\right)}^{B}|\;>c\}). \end{array}$$

We place *T̂P*(*c*), *mean F̂P*(*c*), and *median F̂P*(*c*) into Equation (6) to obtain the *mean FD̂R* and *median FD̂R* for the fixed cut-off value, *c*, as follows

(9) $$mean\;F\hat{D}R=\frac{{\hat{\pi }}_{0}\cdot mean\;\hat{F}P(c)}{\hat{T}P(c)}$$

and

(10) $$median\;F\hat{D}R=\frac{{\hat{\pi }}_{0}\cdot median\;\hat{F}P(c)}{\hat{T}P(c)}.$$

Similarly, the *75th percentile FD̂R* and *90th percentile FD̂R* are defined as

$$75th\;perc.\;F\hat{D}R=\frac{{\hat{\pi }}_{0}\cdot 75th\;perc.\;\hat{F}P(c)}{\hat{T}P(c)}$$

and

$$90th\;perc.\;F\hat{D}R=\frac{{\hat{\pi }}_{0}\cdot 90th\;perc.\;\hat{F}P(c)}{\hat{T}P(c)},$$

respectively. Note that we calculate the ordered statistics *t̄*_(1)_*^b^* ≤ *t̄*_(2)_*^b^* … *t̄*_(_*_g_*_)_*^b^* to determine the cut-off value, *c*, corresponding to the fixed threshold Δ = *t̄*_(_*_i_*_)_ − *t*_(_*_i_*_)_ in SAM (Chu et al. 2005; Tusher et al. 2001). However, in this paper, we determined the cut-off value, *c*, corresponding to the target FDR because the procedure for determining the cut-off value is not relevant to the accuracy of the estimated FDR.

### Simulation studies

We conducted Simulation study 1 to compare the performance of the five statistics, i.e. the Mann-Whitney statistic, Golub’s discrimination score, the Welch *t-*statistic, the *t-*type score, and the variance stabilized *t-*type score. The criterion for examining the performance is the receiver operating characteristic (ROC) curve in which the proportions of both false positives and false negatives are used (Broberg, 2003). Based on the ROC curve, we consider that a test statistic whose ROC curve lies below that of another test statistic has better performance. Simulation study 1 is designed to have 4,000 (*i* = 1, …, 4,000) genes in total, including *s* differentially expressed genes (*i* = 1, …, *s*) and 4,000-*s* nondifferentially expressed genes (*i* = *s* + 1, …, 4,000). Each condition has an equal sample size *N* (*N* = *m* = *n*). For *j* = 1, …, *N*, we generate

$$X_{ij}∼Normal(\mu_{i},{\sigma_{1}}^{2}),i=1,\ldots ,s,$$

$$X_{ij}∼Normal(0.0,{\sigma_{i}}^{2}),i=s+1,\ldots ,4,000,$$

and

$$Y_{ij}∼Normal(0.0,{\sigma_{i}}^{2}),i=1,\ldots ,4,000.$$

Since each true mean of the expression levels of differentially expressed genes is different, we assume a random effect model, i.e. *μ_i_* ∼ *Normal* (1.0, 0.1^2^), *i* = 1, …, *s*. We focus on small sample size experiments under two conditions, and the sample size (*N*) is set as 3, 5, or 10. The number of differentially expressed genes (*s*) corresponding to 1% or 10% is set as 40 or 400, respectively. In the case of constant variance, we set σ*_i_*^2^ = 0.5^2^. We also generate a random standard deviation for each gene from the normal distribution with mean 0.5 and variance 0.1^2^ for random variances case. Additionally, the replication of simulation is set as 1,000, and the ROC curve is drawn using the average of 1,000 values of the estimated proportions of false positives and false negatives.

We also conducted Simulation study 2 to examine the accuracy of the *mean FDR*, the *median FDR*, the *75th percentile FDR*, and the *90th percentile FDR* when the variance stabilized *t*-type score is used. The criterion for examining the accuracy is the scatter plot of the true FDR versus the estimated FDRs. Based on the scatter plot, the line above the diagonal shows overestimation, while that below the diagonal shows underestimation. The simulated data and simulation conditions are the same as those of Simulation Study 1. In the additional conditions, the number of permutations, *B*, is set as 200 in SAM and the proportion of true nondifferentially expressed genes, π_0_, is set as 1 - s/4,000 for each condition in order to estimate the FDR in Equation (6). The scatter plot is drawn using the average of 1,000 values of the true FDR and the each estimated FDR.

### Application to colorectal cancer data

Colorectal cancer (CRC) data were measured for comparing the polysomal RNA from isogenic cell lines established from a CRC patient (http://www.ncbi.nlm.nih.gov/projects/geo/gds/; Provenzani et al. 2006). In the CRC data, 3 cells were derived from a primary tumor and a lymph node metastasis, respectively. Each single RNA sample was subjected to microarray data analysis on Affymetrix DNA chips bearing approximately 22,000 probe sets, which collectively interrogate 14,500 human genes. Details are given in Provenzani et al. (2006). SAM using the three statistics, i.e. the Welch *t*-statistic, the *t*-type score, and the variance stabilized *t*-type score, was applied to this data. The number of permutations, *B*, is set as 200, and the proportion of true nondifferentially expressed genes, π_0_, is estimated by the method of Chu et al. (2005).

## Results

### Results of simulation studies

**Figure 1:**
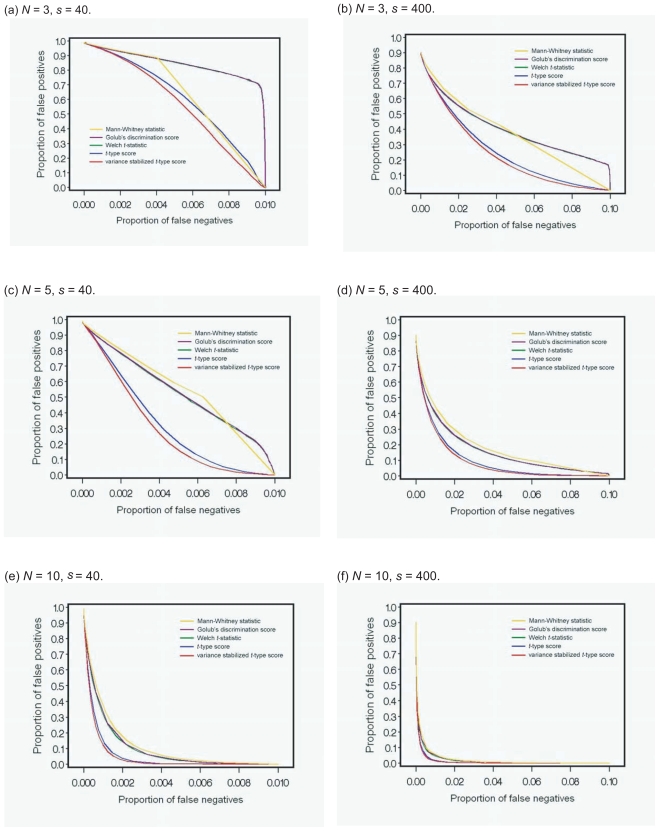
Performance of each test statistic in Simulation study 1.

We discuss only constant variance cases because the ROC curves in Simulation study 1 and the scatter plot in Simulation study 2 of both constant variance cases and random variances cases are almost the same. Figure 1 shows the performance of each test statistic, based on the ROC curve. The *t*-type score and the variance stabilized *t*-type score outperformed the other three test statistics, irrespective of the sample size and the proportion of differentially expressed genes. The difference in performance between them became large when the sample size or the proportion of differentially expressed genes decreased. The variance stabilized *t*-type score outperformed the *t-*type score when *N* = 3 or 5, but it was slightly better than or as good as the *t*-type score when *N* = 10. The difference in the performance between the variance stabilized *t*-type score and the *t*-type score became large when the sample size or the proportion of differentially expressed genes decreased. The performances of the Mann-Whitney statistic, Golub’s discrimination score, and the Welch *t*-statistic are almost same when the sample size is greater than 5.

**Figure 2:**
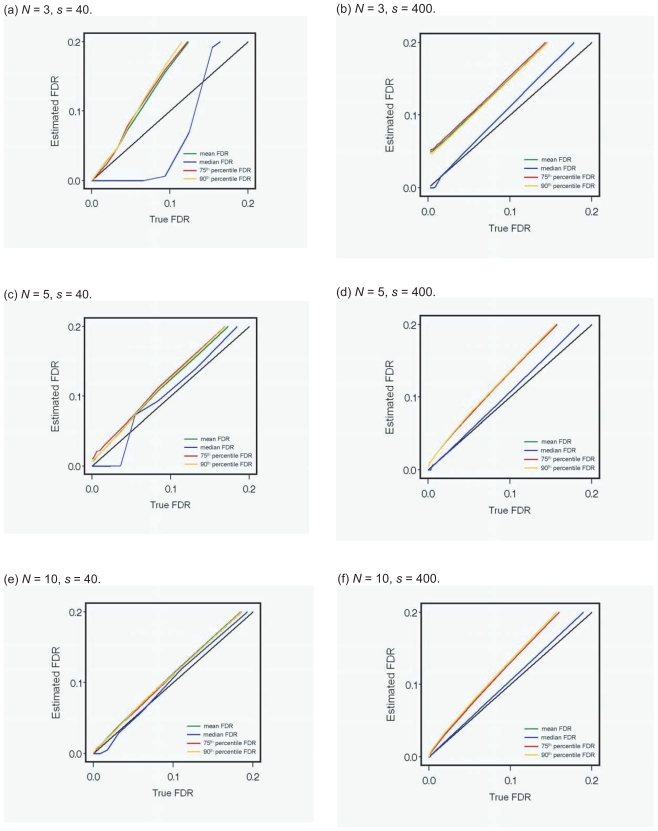
Accuracy of each FDR in Simulation study 2.

Figure 2 shows that the accuracy of the *mean FDR*, *median FDR*, *75th percentile FDR*, and *90th percentile FDR* based on the scatter plot when the true FDR was smaller than 0.2. Each estimated FDR was calculated using the true proportion of nondifferentially expressed genes, π_0_. The biases of the *mean FDR*, *75th percentile FDR*, and *90th percentile FDR* were almost the same, irrespective of the sample size and the proportion of differentially expressed genes. When *s* = 40, the *mean FDR*, *75th percentile FDR*, and *90th percentile FDR* were constantly overestimated, whereas the *median FDR* was overestimated or underestimated depending on the true FDR. In particular, the *median FDR* was underestimated when the true FDR was low. When *s* = 400, the *mean FDR*, *75th percentile FDR*, and *90th percentile FDR* were overestimated, whereas the *median FDR* was almost unbiased.

### Results of colorectal cancer data analysis

**Figure 3:**
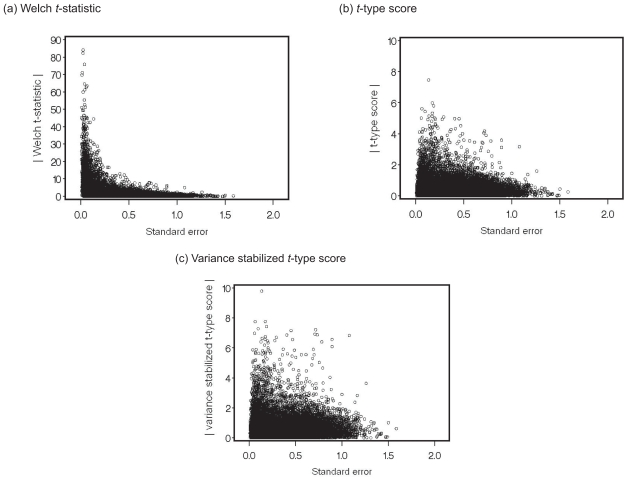
Relationships between the three statistics and the standard error of the Welch *t*-statistic.

Figure 3 shows the relationship between the three statistics, the Welch *t*-statistic, the *t*-type score, and the variance stabilized *t*-type score, and the standard error of the Welch *t*-statistic, $\sqrt{s_{Xi}^{2}/m+s_{Yi}^{2}/n}$, in the CRC data. The results shown in Fig. 3 (a) suggest that the absolute Welch *t*-statistics of the majority of genes became large due to the underestimation of variance, resulting in an increased risk of false positives. In Fig. 3 (b), it appears that the absolute *t-*type score suppressed false positives by controlling the underestimation of variance, and the *t*-type score contributed to the precise identification of differentially expressed genes. Fig. 3 (c) suggested that the variance stabilized *t*-type score controlled the underestimation of variance as well as the *t*-type score and also controlled the overestimation of variance because the ranks of the genes with sample variances that were not small became high when compared with Fig. 3 (b). This result indicates that the overestimation of variance was left uncontrolled in the *t*-type score, while the variance stabilized *t*-type score could control both the overestimation and underestimation of variance in the CRC data. Thus, the variance stabilized *t*-type score made a greater contribution to the precise identification of differentially expressed genes than the *t*-type score.

Table 1 shows the estimated *TP*, the estimated *mean FDR*, and the estimated *median FDR* using the three statistics, the Welch *t*-statistic, the *t*-type score, and the variance stabilized *t*-type score. The estimated proportions of the true nondifferentially expressed genes, π̂_0_, for the Welch *t-*statistic, the *t-*type score, and the variance stabilized *t*-type score were 0.636, 0.646, and 0.606, respectively. Each value of π̂_0_ was almost the same in the CRC data. The estimated number of total positives of the Welch *t*-statistic was extremely large and would contain many false positives. Since the *T̂P* of both the *t*-type score and the variance stabilized *t*-type score was smaller than that of the Welch *t*-statistic, many false positives were suppressed. The *T̂P* of the variance stabilized *t*-type score was larger than that of the *t*-type score because the variance stabilized *t*-type score controlled both the overestimation and underestimation of variance, resulting in a decreased risk of both false positives and false negatives.

The estimated *median FDR* was smaller than the estimated *mean FDR* irrespective of the test statistic. Based on the results of Simulation study 2, the *median FDR* was almost unbiased, whereas the *mean FDR* was overestimated when *N* = 3 and *s* = 400. Therefore, the *median FDR* is recommended as the criterion for identifying differentially expressed genes in the CRC data. When the cut-off value was 2.5, the estimated *median FDR* of the *t*-type score was 0, i.e. 226 genes contained no false positives, while the estimated *median FDR* of variance stabilized *t*-type score was 0.008 when the cut-off value was 2.5, and 469 genes contained hardly any false positives. Probably, several hundreds of genes may be identified as nondifferentially expressed genes when the *t*-type score is used, despite the *t*-type score of such genes is really underestimated due to the overestimation of variance.

## Discussion

### Utility of variance stabilized *t*-type score

In this paper, we devised the variance stabilized *t*-type score using the shrunken sample variances of the James-Stein type and examined its performance. The results of both Simulation study 1 and CRC data analysis revealed the characteristics of the five test statistics, i.e. the Mann-Whitney statistic, Golub’s discrimination score, the Welch *t*-statistic, the *t*-type score, and the variance stabilized *t*-type score, in small microarray experiments. The Mann-Whitney statistic, Golub’s discrimination score, and the Welch *t*-statistic cannot control both the overestimation and underestimation of variance, thereby resulting in an increased risk of both false positives and false negatives. In the case where these test statistics are used, many false positives are identified as differentially expressed genes even if the cut-off value corresponding to a small target FDR is determined to suppress false positives. However, the Mann-Whitney statistic and Welch *t*-statistic can provide the *p* value as another criterion for identifying differentially expressed genes. Since the *t*-type score and the variance stabilized *t*-type score cannot provide the *p* value, we may be able to use the Mann-Whitney statistic or the Welch *t*-statistic if the sample size is sufficiently large. Based on the results of the additional simulation study and a recent study in terms of the usual *t*-statistic (Pan, 2002), the identification of differentially expressed genes using the Welch *t*-statistic is reliable when the sample size is more than 30, irrespective of the proportion of differentially expressed genes.

The basic idea of adding a correction term to the denominator of the Welch *t*-statistic in terms of the *t*-type score had been introduced by Tusher et al. (2001). The utility of a modified *t*-statistic such as the SAM-statistic or the *t*-type score has been demonstrated by some researchers (Baldi and Long, 2001; Broberg, 2003), and we evaluated the utility of the *t*-type score in small microarray experiments. The *t*-type score, which can control the underestimation of variance in particular, showed a better performance than the Welch *t*-statistic irrespective of the sample size because the sample variances are underestimated by chance in small microarray experiments. On the other hand, we noted that the *t*-type score leaves the overestimation of variance uncontrolled. For precise identification of differentially expressed genes, the overestimation of variance should be controlled because both overestimation and underestimation of variance occur simultaneously in actual microarray data analysis.

The shrunken sample variances of the James-Stein type in the variance stabilized *t*-type score borrow information across genes using the James-Stein shrinkage concept. This is useful for identifying differentially expressed genes because the variance is not estimated precisely when the sample size is small. We demonstrated that the variance stabilized *t*-type score was better than or at least as good as the *t*-type score by using simulated and actual data. In particular, the variance stabilized *t*-type score outperformed the *t*-type score when the sample size or the proportion of differentially expressed genes was small, and the relative superiority of the variance stabilized *t*-type score was almost the same when the sample variances were either constant or random. Thus, the variance stabilized *t*-type score is effective and robust for identifying differentially expressed genes in small microarray experiments.

### Characteristics of the *mean FDR* and *median FDR*

The original description of SAM provides the *mean FDR*, while the SAM software of the R package provides the *median FDR*. Because both estimated FDRs are quite different in the actual microarray data analysis, biological researchers are confused with regard to which FDR is a suitable criterion for actual individual microarray data. Indeed, the difference between the estimated *mean FDR* and estimated *median FDR* was approximately 0.1 when the variance stabilized *t*-type score was used in the CRC data analysis. This difference is meaningfully large because approximately 22,000 genes were evaluated simultaneously, although the magnitude of the difference was small. To our knowledge, no studies have been conducted in which the accuracy of the *median FDR* was examined, although some studies have examined the accuracy of the *mean FDR* (Efron et al. 2001; Pan, 2003). The result of Simulation study 2 revealed the characteristics of the four FDRs as determined by SAM. As pointed out by Pan et al. (2003) in terms of the *mean FDR*, the estimated distribution of nondifferentially expressed genes based on the permutation (null distribution) was more dispersed than the distribution of true nondifferentially expressed genes. In other words, the variation of distribution that consists of the estimated number of false positives for the fixed cut-off value in each permutation was very large. The mean of such a distribution became large, resulting in overestimation of the FDR. This disadvantage was exacerbated when the sample size was small or the proportion of differentially expressed genes was large based on the results of Simulation study 2. The *median FDR* was almost unbiased when the proportion of differentially expressed genes was large even if the sample size was small. This feature of the *median FDR*, i.e. the accuracy does not vary according to the sample size, is attractive in small microarray experiments. However, the *median FDR* was underestimated when the true FDR and the proportion of differentially expressed genes was small. The magnitude of underestimation increased when the sample size decreased. The reason for the underestimation of the *median FDR* is that the median of distribution that consists of the estimated number of false positives for the large cut-off value in each permutation becomes very sparse when the sample size or the proportion of differentially expressed genes is small. Specifically, the estimated number of false positives in each permutation becomes almost zero in the case where the large cut-off value is used when the sample size or proportion of differentially expressed genes is small. In such a case, although the true number of false positives is more than 1, the median of the distribution becomes 0, thereby resulting in underestimation of the FDR. Further, through the additional simulation study, we confirmed that the *median FDR* also outperformed the *mean FDR* when *s* = 200, corresponding to 5% as the proportion of differentially expressed genes, and the five FDRs based on SAM using the *t*-type score, show a similar performance to that of the variance stabilized *t*-type score. Our results indicated that we mainly need to make a choice between the *mean FDR* and the *median FDR* based on the estimated proportion of differentially expressed genes. Based on the result of Simulation study 2, we recommend the use of the *median FDR* as the criterion when the estimated proportion of differentially expressed genes is more than 1%, irrespective of the sample size. We also recommend that robustness and reliability be evaluated based on both the *mean FDR* and the *median FDR* when the estimated proportion of differentially expressed genes is less than 1%. In the case of CRC data analysis, we should identify the differentially expressed genes based on the estimated *median FDR*.

### Concluding remarks

We devised the variance stabilized *t*-type score based on the shrunken sample variances and examined its performance, and the accuracy of the *median FDR* by SAM. The utility of the variance stabilized *t*-type score and the characteristics of the *median FDR* were demonstrated through their application to simulated and actual data. Our results indicated that use of the variance stabilized *t*-type score with the *median FDR* by SAM is an effective and robust method for identifying differentially expressed genes when the proportion of differentially expressed genes is more than 1% in small microarray experiments.

## Figures and Tables

**Table 1:** Results of the CRC data analysis.

	Welch *t*-statistic (π̂_0_ = 0.636)			*T*-type score (π̂_0_ = 0.646)			Variance stabilized *t*-type score (π̂_0_ = 0.606)		
Cut-off value	*T̂P*	*Mean FD̂R*	*Median FD̂R*	*T̂P*	*Mean FD̂R*	*Median FD̂R*	*T̂P*	*Mean FD̂R*	*Median FD̂R*
2.0	8221	0.220	0.142	376	0.106	0.012	811	0.115	0.028
2.1	7880	0.211	0.131	338	0.103	0.010	708	0.111	0.025
2.2	7576	0.202	0.121	308	0.102	0.008	629	0.108	0.019
2.3	7294	0.194	0.112	278	0.100	0.005	578	0.103	0.015
2.4	6994	0.186	0.103	251	0.099	0.003	522	0.100	0.010
2.5	6722	0.180	0.097	226	0.098	0.000	469	0.097	0.008
